# Podocyte-specific deletion of miR-146a increases podocyte injury and diabetic kidney disease

**DOI:** 10.3389/fmed.2022.897188

**Published:** 2022-08-18

**Authors:** Xiaobo Li, Ishwarya Venkatesh, Veronica Villanueva, Huiting Wei, Terese Geraghty, Anugraha Rajagopalan, Richard W. Helmuth, Mehmet M. Altintas, Hafeez M. Faridi, Vineet Gupta

**Affiliations:** ^1^Department of Internal Medicine, Drug Discovery Center, Rush University Medical Center, Chicago, IL, United States; ^2^Department of Pathology, The First Affiliated Hospital Sun Yat-sen University, Guangzhou, China; ^3^College of Pharmacy, Chicago State University, Chicago, IL, United States; ^4^Division of Hematology, Oncology and Cellar Therapies, Department of Internal Medicine, Rush University Medical Center, Chicago, IL, United States

**Keywords:** podocytes, MicroRNA, miR-146a, diabetic nephropathy, glomerular disease

## Abstract

Diabetic glomerular injury is a major complication of diabetes mellitus and is the leading cause of end stage renal disease (ESRD). Healthy podocytes are essential for glomerular function and health. Injury or loss of these cells results in increased proteinuria and kidney dysfunction and is a common finding in various glomerulopathies. Thus, mechanistic understanding of pathways that protect podocytes from damage are essential for development of future therapeutics. MicroRNA-146a (miR-146a) is a negative regulator of inflammation and is highly expressed in myeloid cells and podocytes. We previously reported that miR-146a levels are significantly reduced in the glomeruli of patients with diabetic nephropathy (DN). Here we report generation of mice with selective deletion of miR-146a in podocytes and use of these mice in models of glomerular injury. Induction of glomerular injury in C57BL/6 wildtype mice (WT) and podocyte-specific miR-146a knockout (Pod-miR146a^–/–^) animals *via* administration of low-dose lipopolysaccharide (LPS) or nephrotoxic serum (NTS) resulted in increased proteinuria in the knockout mice, suggesting that podocyte-expressed miR-146a protects these cells, and thus glomeruli, from damage. Furthermore, induction of hyperglycemia using streptozotocin (STZ) also resulted in an accelerated development of glomerulopathy and a rapid increase in proteinuria in the knockout animals, as compared to the WT animals, further confirming the protective role of podocyte-expressed miR-146a. We also confirmed that the direct miR-146a target, ErbB4, was significantly upregulated in the diseased glomeruli and erlotinib, an ErbB4 and EGFR inhibitor, reducedits upregulation and the proteinuria in treated animals. Primary miR146^–/–^ podocytes from these animals also showed a basally upregulated TGFβ-Smad3 signaling *in vitro*. Taken together, this study shows that podocyte-specific miR-146a is imperative for protecting podocytes from glomerular damage, *via* modulation of ErbB4/EGFR, TGFβ, and linked downstream signaling.

## Introduction

Diabetes mellitus affects more than 37 million Americans (>11% of the US population) and remains one of the most common medical conditions (1). Progressive glomerular kidney injury due to diabetes mellitus is the leading cause of end-stage renal disease (ESRD) in the US (2). The diabetic glomerulopathy is characterized by loss of glomerular podocytes, glomerular basement membrane (GBM) thickening, segmental glomerulosclerosis, and mesangial expansion. Podocytes are specialized cells located in the Bowman’s capsule of the glomerulus and are essential for the formation and function of the urinary filtration barrier in the kidney. Thus, these cells are vital to a healthy glomerulus and normal kidney function (3, 4). Diabetes results in significant podocyte injury (2, 3), although the exact molecular mechanisms are unclear. Some disease-associated pathways have been elucidated in recent studies (2, 4–12), however, mechanisms that lead to podocyte damage during diabetic nephropathy (DN) pathogenesis are yet to be elucidated. Furthermore, targeted therapeutics are sorely needed in the clinic for those suffering from DN.

MicroRNAs (miRNAs) are a family of small, non-coding regulatory RNAs that are approximately 18–22 nucleotides (nt) in length. Among their key functions, they regulate post-transcriptional expression of their target genes by promoting messenger RNA (mRNA) degradation or suppressing mRNA translation into functional proteins by binding the 3’ untranslated region (UTR) of target mRNAs in a sequence-dependent manner (5). Thus, miRNAs play a vital role in regulating cell biological functions. Like protein expressing mRNAs, the expression of miRNAs is also regulated in a tissue specific fashion. Different miRNAs are expressed in all stages of kidney development and are also differentially regulated in the glomeruli in response to various external stimuli, indicating their involvement in disease pathogenesis (6). miRNAs are essential in podocyte homeostasis, as conditional deletion of miRNA processing enzymes Drosha and Dicer results in significant proteinuria and accelerated glomerular injury (7–9). Similarly, deletion of specific miRNAs in podocytes results in increased proteinuria and glomerulosclerosis (7). Expression levels of multiple miRNAs change in early DN, resulting in a change in expression of a number of DN associated genes and pathways and playing an important role in the pathophysiology of diabetic glomerular injury (10).

MicroRNA-146a (miR-146a) is a negative regulator of the pro-inflammatory signaling in myeloid cells and thus a key molecular brake on the inflammatory innate immune cell responses (11, 12). It also modulates key adaptive immune cell functions and plays an important role in hematopoiesis and cancer cell proliferation, *via* targeting a different set of genes (11, 13). miR146a is also highly expressed in the podocytes and in all other in types of cells in the glomeruli, suggesting that it has important homeostatic and regulatory roles in podocytes, including regulating kidney function during diabetic injury (14–16). Myeloid cell-expressed miR-146a was recently shown to increase in expression in murine DN, where it plays an anti-inflammatory role by suppressing proinflammatory cytokines in macrophages (17). Conversely, we previously found that the glomerular miR-146a levels were dramatically reduced in the kidney sections of diabetic patients (18), and that the level of miR-146a expression in the kidneys inversely correlated with proteinuria in the patients, suggesting that podocytic miR-146a plays a protective role in DN. A recent study with 460 subjects (300 cases and 160 controls) also confirmed that there exits an inverse association of blood miR-146a levels with diabetes and its complications (19). Furthermore, miR-146a levels were found to be down-modulated in the kidneys of diabetic rats and mice, suggesting that it plays a reno-protective role (20). We previously showed that the absence of miR-146a in the global miR-146a knockout animals (miR-146a^–/–^) increased susceptibility of these animals to diabetic kidney disease, *via* hyperglycemia-induced podocyte damage (18). However, given that these prior studies used global miR-146a^–/–^ animals, the studies were not able to provide direct evidence of the podocyte specific role of miR-146a.

To address this, we report here generation of podocyte-specific miR-146a^–/–^ animals (Pod-miR146a^–/–^) to study the functional consequences of the absence of miR-146a specifically in the glomerular podocytes. We find that the Pod-miR146a^–/–^ mice mimic the diabetic glomerular injury observed in the global miR-146a^–/–^ animals (18). Additionally, given that miR-146a directly targets tyrosine receptor kinase ErbB4, we again find that the absence of miR146a results in increased ErbB4 expression and signaling, thus driving a podocyte-damaging feed-forward loop. Altogether, the results presented here confirm the role of podocyte-specific miR-146a in protecting podocytes from damage during DN pathogenesis, *via* control of ErbB4 and downstream signaling.

## Results

### Animals with podocyte-specific deletion of miR-146a are more sensitive to lipopolysaccharide and nephrotoxic serum-induced renal injury

**FIGURE 1:**
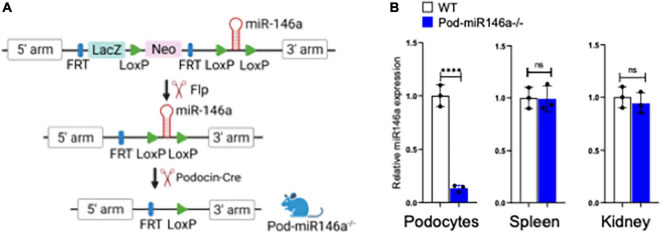
Generation of podocyte-specific miR-146a deleted mice. **(A)** Schematic showing steps used in the generation of podocyte-specific miR146a knockout mice by crossing miR146a*^flox/flox^* mice with Flp-mice and subsequently with podocin-cre mice. **(B)** miR-146a expression levels measured by quantitative RT-qPCR from primary podocytes, spleen and whole kidney isolated from WT or Pod-miR146a mice. Statistics were performed using the Student’s *t*-test. Data shown are mean ± SEM (*n* = 3). ***p < 0.0001; ns, no significant difference.

Healthy podocytes abundantly express miR-146a, where it plays an important role in cellular health and function (14, 15). We previously showed that miR-146a levels significantly decreased in the glomeruli of diabetic patients and that the decrease was associated with progressively increasing kidney damage (18). Furthermore, using animals with global deletion of miR-146a (miR146a^–/–^), we also showed that the absence of miR-146a accelerated the progression of diabetic nephropathy (DN) in the knockout animals. However, miR-146a is also highly expressed in the immune cells, where it suppresses NFκB signaling pathways and has been shown to function as an anti-inflammatory miRNA (12, 13). Given our previous data with DN patient biopsies and the DN models in miR-146a^–/–^ mice, we hypothesized that the podocyte-expressed miR-146a plays an essential role in protecting glomeruli from injury. To unambiguously explore this role of miR-146a, we developed a novel, podocyte-specific miR146a deleted transgenic mouse (referred to as Pod-miR146a^–/–^) using published protocols (21) (Figure 1A). Briefly, we first crossed C57BL/6 miR146a*^fl/fl^* mice containing floxed sites around the miR146a exon with Flp-recombinase expressing mice to remove the selection cassette containing LacZ and neomycin. Next, we crossed homozygous progeny with Podocin-Cre recombinase-expressing mice (22), which recognize the loxP sites surrounding the miR146a gene and only cut these sites in cells under the control of the Podocin promoter (which is highly and selectively in podocytes), thus selectively deleting miR146a specifically in podocytes to obtain Pod-miR146a^–/–^ mice. The resulting mice were backcrossed >6 times before use in experiments here. Quantitative RT-PCR (qRT-PCR) based analyses of isolated podocytes showed an almost complete loss of miR-146a expression, as compared to miR-146a levels in cells from the wild type C57BL/6 wild-type (WT) littermates, whereas miR-146a expression in spleen and whole kidney showed no significant difference, as compared to the WT animals (Figure 1B). These data confirm successful deletion of miR-146a in podocytes.

**FIGURE 2:**
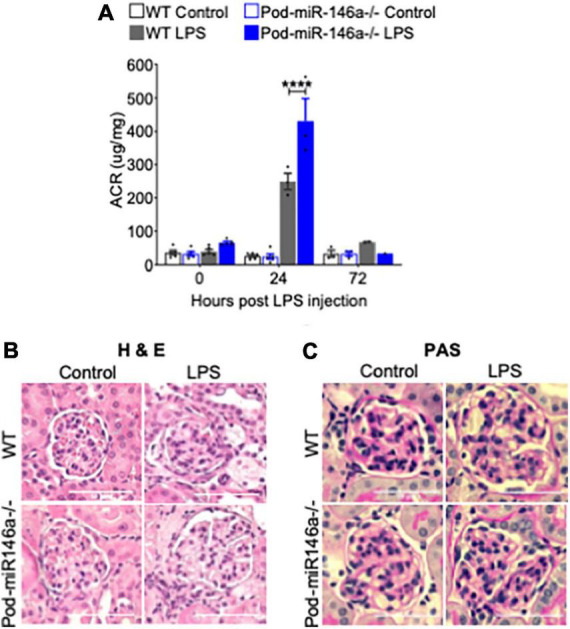
*Pod-miR-146a mice show increased impairment of kidney function in response to LPS.* **(A)** Graph showing the albumin to creatinine ratio (μg/mg) in the urine of WT (gray bars) and Pod-miR-146a (blue bars) mice after 0, 24, and 72 h post administration of either vehicle (open bars) or LPS (shaded bars). Statistics were performed using two-way ANOVA. Data shown are mean ± SEM (*n* = 5). ****p < 0.0001. **(B,C)** Representative images showing histochemical analyses with hematoxylin-eosin (H&E), periodic acid-Schiff (PAS) staining of kidney tissues from 24 h post LPS treatment. Scale bar, 50 μm.

In order to evaluate the effect of podocyte-specific miR146a deletion in models of glomerular injury, we examined its role in low-dose lipopolysaccharide (LPS) induced glomerular injury model of focal segmental glomerulosclerosis (FSGS) (23). Expectedly, LPS administration into WT mice resulted in a strong induction of albuminuria after 24 h compared with vehicle controls (Figure 2A). LPS administration into the Pod-miR146a^–/–^ animals produced a significantly higher rise in albuminuria as compared to the LPS-treated WT animals, suggesting the lack of miR-146a in podocytes exacerbates glomerular injury. Histochemical analyses confirmed significant glomerular damage in the LPS-treated groups compared to controls (Figures 2B,C). These results suggest that podocyte-specific miR146a is protective of acute glomerular injury.

**FIGURE 3:**
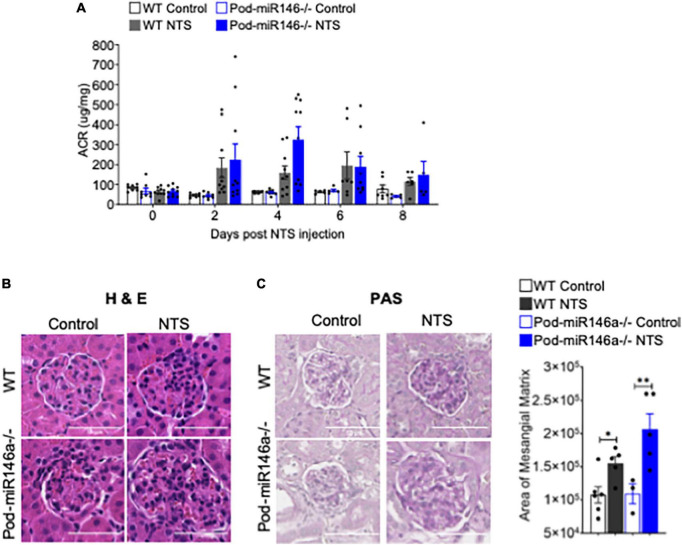
Increased albuminuria in Pod-miR-146a^–/–^ mice after NTS treatment. **(A)** Graph showing the albumin to creatinine ratio (μg/mg) in the urine of WT (gray bars) and Pod-miR-146a (blue bars) mice post administration of either vehicle (open bars) or NTS (shaded bars) at various time-points, as indicated. Statistics were performed using two-way ANOVA. Data shown are mean ± SEM (*n* = 5). Representative images showing histochemical analyses with **(B)** H&E and **(C)** PAS staining of kidney tissues from each of the four groups of animals from 8th day post NTS treatment. Scale bar, 50 μm. Graph showing quantified mesangial matrix from the PAS-stained sections. Statistics were performed using the Mann-Whitney test. Data shown are normalized to the level of staining in control tissue and are mean ± SEM (*n* = 3). *p < 0.05; **p < 0.01.

We further investigated the role of miR146a in glomerular injury using an established model of anti-glomerular basement membrane (anti-GBM) nephritis (24). Administration of nephrotoxic serum (NTS) into WT and Pod-miR146a^–/–^ mice resulted in progressively increased albuminuria, with the Pod-miR146a^–/–^ animals showing significantly worse disease and peak proteinuria at 4 weeks post NTS administration (Figure 3A), suggesting that miR146a plays an important role in protecting glomeruli from chronic injury. Histochemical analyses further confirmed significantly higher glomerular damage, including increased mesangial matrix expansion, in the NTS-treated Pod-miR146a^–/–^ mice (Figures 3B,C). Altogether, these data show that podocytic miR146a is protective and its loss exacerbates glomerular injury in acute and chronic settings.

### Diabetic glomerular injury is exacerbated in Pod-miR146a^–/–^ animals and is reduced by erlotinib

**FIGURE 4:**
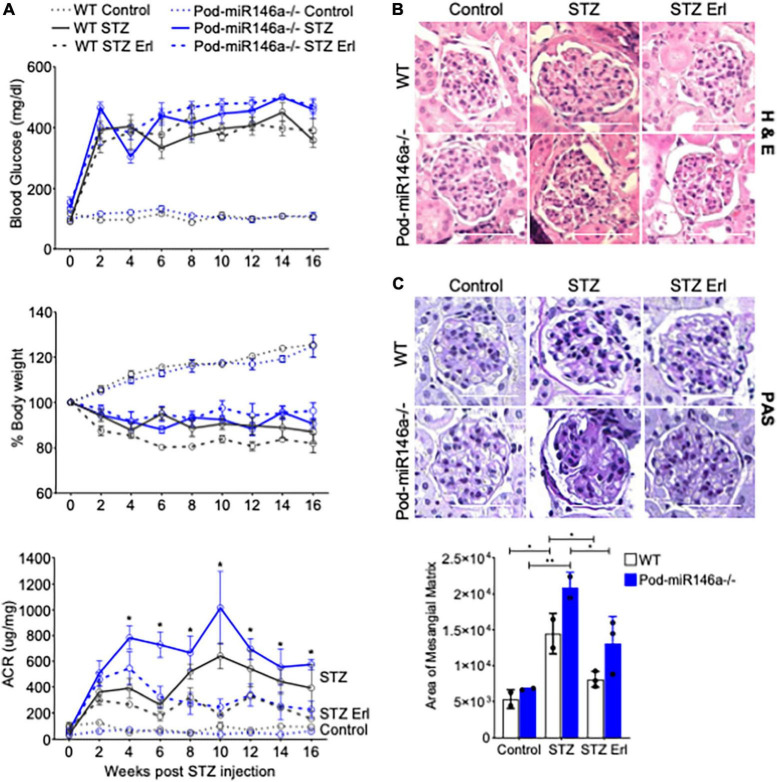
STZ accelerates glomerular injury in Pod-miR-146a^–/–^ mice that is attenuated by erlotinib. **(A)** (Top) Graph showing levels of hyperglycemia in various animals, as measured by serum glucose levels (mg/dL) in each of the six groups, as indicated, post STZ administration and at various time-points, as indicated. Data shown are mean ± SEM (*n* = 5). Treatment with erlotinib starting at 4 weeks after STZ-induction did not result in any change in level of hyperglycemia in either strain. Blood glucose levels remained unchanged in the non-STZ treated mice; (Middle) Graph showing levels of weight loss upon in various animals in each of the six groups, as indicated, post STZ administration and at various time-points, as indicated. Data shown are mean ± SEM (*n* = 5). WT and Pod-miR146a animals displayed equal levels of weight loss upon STZ-induced hyperglycemia that was unaffected by treatment with erlotinib; (Bottom) Graph showing the albumin to creatinine ratio (μg/mg) in the urine of mice from each of the six groups, as indicated, post STZ administration and at various time-points, as indicated. Statistics were performed using one-way ANOVA. Data shown are mean ± SEM (*n* = 5). *p < 0.05. **(B)** Representative images showing histochemical analyses with H&E staining of kidney tissues from each of the six groups of animals at end point. Scale bar, 50 μm. **(C)** Representative images showing histochemical analyses with PAS staining of kidney tissues from each of the six groups of animals at end point. Scale bar, 50 μm. Graph showing quantified mesangial matrix from the PAS-stained sections. Data shown are normalized to the level of staining in control tissue and are mean ± SEM (*n* = 3). *p < 0.05; **p < 0.01.

To further investigate the effect of podocyte-specific miR146a deletion on glomerular function *in vivo*, we induced hyperglycemia in WT or Pod-miR146a^–/–^ animals using published STZ protocols (25). STZ treatment resulted in induction of hyperglycemia and body weight decline in WT animals and to a similar extent in the Pod-miR146a^–/–^ mice (Figure 4A), confirming our recent findings with the global miR-146a^–/–^ mice (17, 18). Both strains also showed diabetes induced increase in albuminuria, although, the WT animals showed a slower increase (25–27), whereas the Pod-miR146a^–/–^ mice showed a more rapid increase (as soon as 6 weeks post STZ injection) and significantly higher albuminuria level over time (Figure 4A), in line with our recent report with the global miR-146a^–/–^ (18) and suggesting that miR-146a is critical for podocyte health and that its loss in podocytes greatly accelerates glomerulopathy *in vivo*.

**FIGURE 5:**
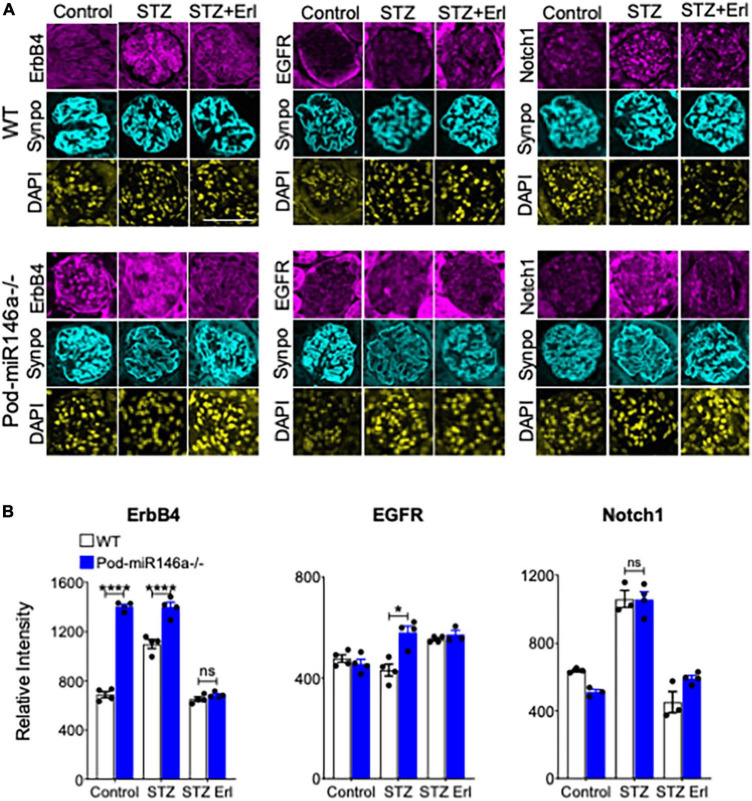
Immunofluorescence imaging-based analyses of glomerular sections shows erlotinib administration protects WT and Pod-miR-146a^–/–^ mice from STZ injury via reduction in ErbB4 and EGFR. **(A)** Representative confocal microscopy images of immunofluorescently labeled glomeruli from WT (top three panels) and Pod-miR146a (bottom three panels) mice treated with vehicle alone (Control), with STZ and vehicle (STZ) or with STZ and erlotinib (STZ Erl). Tissue sections were imaged after staining with DAPI (nuclear marker) and antibodies against ErbB4, EGFR, Notch-1 and Synaptopodin (Synpo, podocyte marker) (as indicated). Scale bar, 50 μm. **(B)** Bar graphs showing quantification of relative glomerular signal intensity of ErbB4, EGFR and Notch-1 in tissue samples from A. Statistics were performed using two-way ANOVA. Data shown are mean ± SEM (*n* = 5/group). *p < 0.05; ***p < 0.001; ns, no significant difference.

ErbB4 (v-erb-b2 avian erythroblastic leukemia viral oncogene homolog 4, also known as HER4) is a tyrosine kinase receptor that is a member of the epidermal growth factor receptor (EGFR, also known as ErbB) family of receptors (28). ErbB4 often heterodimerizes with EGFR to form a functional receptor. ErbB4 mRNA is a direct molecular target of miR-146a, and binding of miR-146a to ErbB4 mRNA targets ErbB4 for degradation (29–32). Previously, we observed that ErbB4/EGFR expression and signaling was upregulated in the diabetic miR-146a^–/–^ animals and that blockade of this pathway with clinically available ErbB4/EGFR inhibitor erlotinib was therapeutic, and erlotinib treatment reduced ErbB4 and EGFR expression and signaling (18, 33). To determine if this pathway was also therapeutic in the context of podocyte-specific miR-146a deletion, we also administered erlotinib to a group of diabetic WT and Pod-miR146a^–/–^ animals. Results show that erlotinib treatment significantly reduced the level of albuminuria in both the diabetic WT and Pod-miR146a^–/–^ animals (Figure 4A), without affecting the increased hyperglycemia or decreased body weight, as expected. This suggests that podocytic miR-146a protects cells *via* suppression of the ErbB4/EGFR pathway. Histopathologic analyses of kidney sections showed significant mesangial sclerosis in the glomeruli of the diabetic WT and Pod-miR146a^–/–^ animals, and that erlotinib treatment significantly protects glomeruli from injury (Figures 4B,C). Immunofluorescence staining of the kidney sections showed a significant upregulation of ErbB4 in the glomeruli of non-diabetic Pod-miR146a^–/–^ at baseline, as compared to the WT animals, suggesting that deletion of miR-146a in podocytes results in increased expression of its direct molecular target ErbB4 (Figures 5A,B). Data showed an increase in ErbB4 in podocytes, as visualized by colocalization of ErbB4 with synaptopodin *via* immunofluorescence (Supplementary Figure 1). Interestingly, no increase was observed for EGFR or Notch-1, unlike our previous findings with the global miR-146a^–/–^ mice (18). Expectedly, STZ-induced diabetic injury resulted in significant increase in the expression of all three markers ErbB4, EGFR and Notch-1 in the diseased glomeruli. Additionally, treatment with kinase inhibitor erlotinib, which suppresses the kinase activity and a feed-forward suppression of kinase expression, showed significant reduction in the expression level of all three harmful proteins. Immunofluorescence imaging-based quantification of podocyte numbers also showed that STZ treatment resulted in a significant reduction in podocytes in both strains and that erlotinib significantly protected both strains from the loss of podocytes (Supplementary Figure 2).

**FIGURE 6:**
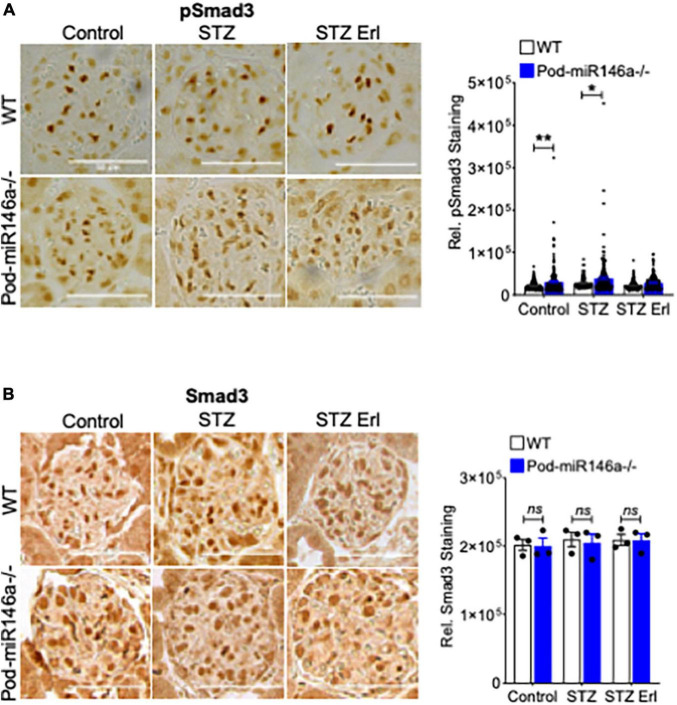
*Increase in glomerular pSmad3 levels by STZ treatment is suppressed by erlotinib.* **(A)** Representative immunohistochemical images of glomeruli stained with an antibody against pSmad3 from WT (top row) and Pod-miR146a mice (bottom row) treated with vehicle alone (Control), with STZ and vehicle (STZ) or with STZ and erlotinib (STZ Erl). Scale bar, 50 μm. Graph on the right shows quantification of pSmad3 positive cells per glomeruli in these samples. Statistics were performed using two-way ANOVA. Data shown are mean ± SEM (*n* = 3). *p < 0.05, **p < 0.01. **(B)** Representative immunohistochemical images of glomeruli stained with an antibody against total Smad3 from WT (top row) and Pod-miR146a mice (bottom row) treated with vehicle alone (Control), with STZ and vehicle (STZ) or with STZ and erlotinib (STZ Erl). Scale bar, 50 μm. Graph on the right shows quantification of total Smad3 positive cells per glomeruli in these samples. Statistics were performed using two-way ANOVA. Data shown are mean ± SEM (*n* = 3). ns, no significant difference.

Transforming growth factor beta 1 (TGFβ1), a member of the TGFβ superfamily, is a central regulator of diabetic glomerular injury and kidney fibrosis. TGFβ also mediates podocyte damage and diabetic glomerular injury in diabetes (34, 35). TGFβ1 imparts its intracellular effects by binding and activating TGFβ receptors (TGFBR), increasing phosphorylation and activation of the downstream Smad2/3 and MAPK signaling pathways (34–37). Given our previous finding that the TGFβ1 levels and signaling were increased in the diabetic miR-146a^–/–^ podocytes (18), we investigated pSmad3 levels in the tissues of the animals here. Immunohistochemical (IHC) staining of kidney sections from untreated WT and Pod-miR-146a^–/–^ mice showed a significant upregulation of pSmad3 staining under basal conditions in the glomeruli (no change in total Smad3) (Figures 6A,B). Additionally, STZ-induced diabetic injury resulted in further, significant increase in the expression of pSmad3 in the diseased glomeruli, and erlotinib treatment significantly suppressed the pSmad3 levels.

Taken together, these data suggest that ErbB4, a direct target of miR-146a, and the ErbB4/EGFR pathway are upregulated in the glomeruli of miR-146a-deficient and in diabetic animals. It also shows a basally upregulated TGFβ signaling pathway, that, together, predispose cells for injury. Podocyte-specific deletion of miR146a also results in worse diabetic glomerulopathy. Finally, our data strongly show that blocking ErbB4/EGFR signaling *via* erlotinib is therapeutic.

### ErbB4 and its downstream signaling components show basal elevation in Pod-miR146a^–/–^ podocytes

**FIGURE 7:**
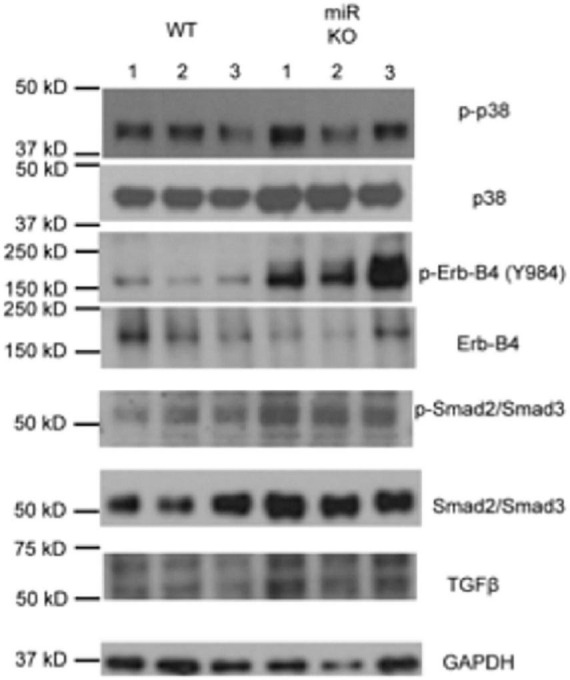
Deletion of miR146a in podocytes upregulates ErbB4/TGFβ /Smad3 signaling. Immunoblot analysis of various phosphorylated (p-) and total proteins in the lysates from primary podocytes isolated from the WT and Pod-miR146a mice. Data presented is from three independent samples from each group. GAPDH was used as the loading control. Relative position of the molecular weight markers is shown on the left.

Next, we used western blot (WB) analysis of lysates from isolated WT and Pod-miR146a^–/–^ primary podocytes to investigate levels of ErbB4, a direct target of miR-146a, and TGFβ signaling in these cells under basal conditions. The analyses showed increased ErbB4 expression as well as elevated levels of phospho-ErbB4 in the miR-146a deleted cells (Figure 7 and Supplementary Figure 3), confirming the tissue immunofluorescence staining findings above. TGFβ1 is a central mediator of glomerular injury and fibrosis in DN *via* activation of mitogen activated protein kinase (MAPK) and Smad-based signaling pathways (38). WB analyses also showed a basal increase in phospho-p38 MAPK and increased levels of TGFβ1 and phospho-Smad2/3, suggesting that absence of miR-146a basally increases the harmful TGFBR/Smad signaling and pre-disposes podocytes to injury. Exact molecular details of how increased ErbB4/EGFR increases TGFb1 levels are not currently known and will need to investigated in the future.

## Discussion

MicroRNAs (miRNAs) are a family of small regulatory RNAs that are central mediators of posttranscriptional repression in mammalian cells, thereby providing finer control of gene expression in cells (5). Thus, changes in expression of various miRNAs results in gene expression changes in cells, leading to different phenotypic outcomes. MicroRNA-146a (miR-146a) is a master regulatory RNA in myeloid cells, where it regulates TLR-dependent pro-inflammatory signaling *via* an NFκB-dependent negative feedback loop (11–13, 39). Recent studies have also shown that this miRNA is expressed widely in multiple types of mammalian tissues, where it regulates expression of target genes. We previously showed that miR-146a expression is significantly reduced in the glomeruli of diabetic patients, and that it negatively correlates with proteinuria in these patients (18). We also showed, using mice with global deletion of miR-146a, that the absence of miR-146a in podocytes drives podocyte damage *via* de-repression of its target genes ErbB4 and Notch-1. However, given our use of global miR-146a^–/–^ animals, it wasn’t clear of the podocyte-expressed miR-146a was the driver of the observed phenotypes. Here, we addressed this by generating mice with podocyte-specific deletion of miR-146a (Pod-miR146a^–/–^) and using these animals in models of kidney disease.

**FIGURE 8:**
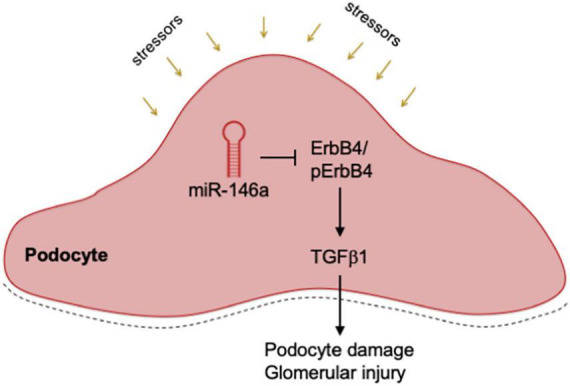
Mechanistic model. A diagram showing a mechanistic working model. Podocyte expressed miR-146a represses expression of ErbB4 and Notch-1 during homeostatic conditions, thereby controlling the ErbB4/EGFR and TGFβ 1 signaling pathways. Various external stressors or deletion of miR-146a result in de-repression of ErbB4 and Notch-1, thereby driving the harmful ErbB4/EGFR signaling and inducing TGFβ 1. An autocrine feed-forward loop via TGFβ 1 induces the downstream TGFR/Smad3 signaling, that result in podocyte damage, glomerular injury and proteinuria.

Our studies show that the Pod-miR146a^–/–^ are more sensitive to induction of kidney injury. We found that administration of LPS induced a significantly higher level of proteinuria in the Pod-miR146a^–/–^ mice as compared to the C57BL/6 WT mice. Similarly, administration of NTS also resulted in significantly higher level of proteinuria and glomerular injury in the Pod-miR146a^–/–^ mice. Additionally, as with the global miR-146^–/–^ animals (18), induction of hyperglycemia *via* STZ administration produced a significantly higher level of proteinuria and increased glomerular sclerosis in the Pod-miR146a^–/–^ animals. Given our previous findings that miR-146a suppresses expression of harmful ErbB4 gene expression, immunofluorescence imaging of the kidney sections confirmed basal increase in expression levels of ErbB4 in the otherwise healthy animals, suggesting that absence of miR-146a results in induction of ErbB4/EGFR pathway. WB based analyses of isolated primary podocytes also confirmed a basal increase in ErbB4 in the cells from Pod-miR146a^–/–^ mice. Finally, previous studies had shown that ErbB4/EGFR signaling results in increased TGFβ-Smad pathway. WB with Pod-miR146a^–/–^ podocytes also confirmed a basal increase in this pathway. Currently, in the absence of direct molecular evidence, it is unclear as to how blockade of ErbB4/EGFR *via* erlotinib suppresses Smad3 activation. We speculate that TGFβ, *via* an autocrine mechanism, elicits the EGFR-Smad3 pathway. Future studies are needed to further delineate the exact molecular mechanisms. Collectively, these data confirm that miR-146a in podocytes has a protective role and that its absence sensitizes podocytes to injury, *via* upregulation of ErbB4/EGFR pathway and induction of TGFβ signaling (Figure 8).

Several recent studies also highlight the role of miR-146a in protecting patients and animals from diabetic end-organ injury. A number of studies, including ours (18), show that reduced miR-146a levels are associated with injury. For example, serum miR-146a levels inversely and independently correlate with chronic complications of diabetes, including cardiovascular disease (CVD) and diabetic retinopathy, in a cohort of patients with type 1 diabetes (19). Similarly, serum miR-146a levels show significant age-related decline in type 2 diabetics and show inverse correlation with creatinine (40). Furthermore, miR-146a was also found to be present in urinary exosomes in hypertensive patients and its levels were reduced in patients with albuminuria (41). Similarly, miR-146a^–/–^ mice showed age-dependent development of immune complex glomerulonephritis (42) and urinary exosomal miR-146a levels were significantly reduced in lupus nephritis patients and has been proposed as a disease biomarker (43). Conversely, there are other studies that suggest that increased miR-146a expression is associated with diabetic kidney injury (17, 44). However, all studies conclude that miR-146a has a protective role, albeit *via* a different mechanism, perhaps because it is widely expressed in many cell types and tissues, thus displaying multi-factorial effects.

Over-expression of miR-146a has also been shown to afford protection from diabetic kidney injury. STZ-induced diabetes in rats reduced expression of miR-146a in the kidney and the heart tissues, and exposure of endothelial cells to high glucose downregulated miR-146a in these cells (20). Furthermore, intravitreal administration of miR-146a mimic restored retinal miR-146a levels. STZ-induced hyperglycemia produced down-regulation of miR-146a in the kidneys of WT mice and resulted in increased kidney and retinal injury, and transgenic mice overexpressing miR-146a in endothelial cells are protected from diabetic retinal and kidney injury (45). Collectively, the published studies and our studies presented here show that miR-146a plays an important, homeostatic role in protecting kidney from injury and that such protection is mediated *via* many different cells present in the kidney. Using the podocyte-specific miR-146a deleted animals, we conclusively show that podocyte-expressed miR-146a is protective.

## Materials and methods

### Materials

Cell culture reagents were purchased from Thermo Fisher Scientific (Waltham, MA). Rat tail collagen I was purchased from Sigma (St. Louis, MO) and mouse recombinant interferon-γ was obtained from Cell Sciences (Canton, MA). Erlotinib was purchased from LC Laboratories (Woburn, MA). The polyclonal goat anti-synaptopodin antibody (P-19) and rabbit anti-ErbB4 antibody (C-18) were purchased from Santa Cruz (Santa Cruz, CA), rabbit anti-EGFR (06-847) from Millipore (Darmstadt, Germany), polyclonal rabbit anti-Notch1 (100-401-407) from Rockland (Limerick, PA), rabbit anti-podocin antibody from Sigma, anti-cleaved-Notch1 antibody (Val-1744) and anti-GAPDH (6C5) antibodies were from Abcam (Cambridge, MA), the anti-EGFR antibody (D38B1), anti-p-EGFR (Tyr-845) antibody, anti-p-EGFR (Tyr-1068) antibody (D7A5), anti-p-EGFR (Tyr-1173) antibody (53A5), anti-p-ErbB4 antibody (Tyr- 984), anti-ERK antibody (137F5), anti-p-ERK antibody (Thr-202/Tyr-Y204), anti-p38 antibody, anti-phospho-p38 (Thr-180/Tyr-182) antibody (28B10), anti-Smad2/Smad3 antibody, anti-p-Smad2 (Ser-465/467)/Smad3 (Ser-423/425) antibody (D27F4), and anti-TGF-β1 antibodies were purchased from Cell Signaling (Danvers, MA).

### Animals

Animal care and procedures were approved by the Institutional Animal Care and Use Committee (IACUC) at Rush University Medical Center and were performed in accordance with institutional guidelines. The C57BL/6J wild-type (WT), and miR-146a*^fl/fl^* (034342) mice (21) were purchased from the Jackson Laboratory (Bar Harbor, ME). Flp recombinase and Podocin-Cre mice were generously provided by the laboratory of Dr. Jochen Reiser (Rush University Medical Center). Generation of podocyte specific miR-146a deleted mice (Pod-miR146a^–/–^) was achieved by first crossing miR-146a*^fl/fl^* mice with Flp recombinase expressing mice to delete the selection cassette. Subsequently, the homozygous progeny were crossed with Podocin-Cre recombinase-expressing mice to delete miR-146a selectively in podocytes (22) (Figure 1A). Knock-out of miR-146a were confirmed by genotyping.

### Blood glucose measurement and urinary albumin and creatinine assays

Blood glucose measurement and urinary albumin and creatinine assays were performed as described previously (18). Briefly, blood glucose was measured from tail vein blood by using a FreeStyle Freedom lite glucometer (Abbott, Abbott Park, IL). For urinary albumin and creatinine measurements, spot urine samples were collected non-invasively from mice. Urinary albumin and creatinine concentrations were measured using a mouse albumin ELISA (Bethyl Laboratories, Montgomery, TX) and a creatinine assay (Exocell, Philadelphia, PA), respectively. Subsequently, protein concentrations and urine albumin-to-creatinine ratios were calculated.

### Primary podocyte isolation

Primary mouse podocytes were isolated as previously published (18). Briefly, mice were anesthetized and perfused with HBSS containing DynaBeads (Invitrogen, #14013). Mouse kidneys were collected from 8- to 16-week-old mice and minced into small pieces and placed in digestion buffer containing collagenase A (Roche) and DNase I (Sigma) and incubated shaking for 30 min at 37^°^C. After passing the digested kidneys twice through a 100 μm cell strainer (Thermo Fisher Scientific), they were spun down (200 × g for 5 min). After resuspending the beads (attached to kidney glomeruli), they were magnetically separated out of solution, washed, counted, and placed in a collaged-coated petri dish with RPMI 1640 medium containing 10% FBS. Podocytes were allowed to grow in culture for 4–5 days before use in downstream analyses (e.g., lysed in Trizol for RNA analysis).

### Immunofluorescence

Immunofluorescence staining of EGFR, Notch-1, ErbB4 and synaptopodin mouse kidney tissues were performed as described previously (18). Briefly, mouse kidney tissues from the different treatment groups from WT and Pod-miR146^–/–^ mice were fixed in formalin and embedded in paraffin for further processing. Tissue sections of 4 μm thickness were deparaffinized and hydrated through xylenes and graded alcohol series before antigen retrieval with citrate buffer (Vector Labs, #H-3300). Sections were incubated in 0.2% Triton-X for 10 min at RT, washed, and incubated with the blocking buffer (4% FBS, 4% BSA and 0.4% fish gelatin in PBS) for 1 h at RT. For EGFR, Notch-1 and ErbB4 staining, sections were then incubated with primary antibodies—Rabbit anti-mouse EGFR (Millipore, #06847), mouse anti-mouse ErbB4 (Santa Cruz, #sc-8050) and Rabbit anti-mouse Notch-1 (Rockland, #100-401-407) in the blocking buffer at 4^°^C, overnight. After incubation, sections were washed and incubated with 0.3% hydrogen peroxide for 30 min at RT to block endogenous peroxidase activity. Sections were then incubated with blocking buffer containing HRP labeled secondary antibody polymer (Vector Labs, #MP7401 or #MP-7402) for 30 min at room RT. Sections were washed, incubated with TSA reagent (Akoya Biosciences, #NEL744001KT) for 10 min, washed and stained with DAPI for 3 min at RT. For synaptopodin staining, serial sections were processed and blocked as described earlier. Sections were then incubated with anti-synaptopodin antibody (Santa Cruz, #sc-515482) and incubated overnight at 4°C. Sections were subsequently washed and incubated with goat anti-mouse AF488 (Thermo Fisher Scientific, #A-11001). Glomeruli from the stained tissues were imaged with a Zeiss 700 LSM confocal microscope (Zeiss) and the images were quantified and analyzed using Image J software (NIH, Bethesda, MD).

### Tissue histochemical staining

Mouse kidneys were harvested after PBS wash. One section of the removed kidney was fixed in 10% formalin and embedded in paraffin and another part was immediately snap frozen in OCT embedding compound on liquid nitrogen and stored at 80^°^C. Paraffin-embedded sections (4 μm) were stained with hematoxylin-eosin (H&E), periodic acid-Schiff (PAS), or Masson’s trichrome. Tissue processing, including fixation, dehydration, embedding, and histochemical staining was performed at University of Illinois at Chicago (UIC) Research Histology and Tissue Imaging Core. Stained slides were blindly evaluated by an experienced pathologist and scanned using Aperio software (Leica, Buffalo Grove, IL). Fibrosis was indicated as a percent of tissue area stained blue with Masson’s trichrome. Quantification for glomerular volume and mesangial expansion was performed according to published methods using ImageJ software (NIH, Bethesda, MD) (46). Glomerular expression of pSmad3 and Smad3 was analyzed by regular immunohistochemical staining using pSmad3 antibody 1:200 dilution, (Abcam, 52903) and Smad3 antibody 1:200 dilution (Proteintech, 25494-1-AP). Briefly, tissue sections were deparaffinized in xylene and rehydrated through descending concentrations of ethanol and subjected to antigen retrieval by steam heating in an acidic pH solution (Citrate-based, Vector Laboratories). Sections were incubated in 0.3% hydrogen peroxidase in water for 30 min and were blocked (4% FBS, 4% BSA, 0.4% fish gelatin) at room temperature for 1 h. Subsequently, sections were incubated with primary antibodies at 4^°^C overnight, followed by washing and incubation with appropriate secondary HRP-labeled secondary antibody polymer (Vector Labs) for 30 min at room temperature. Signal development was accomplished by using the DAB substrate kit (Vector Labs). pSmad3 and Smad3 quantification was done by counting marker positive cells in four independently, randomly chosen areas analyzed at 20x using a Zeiss 700 LSM confocal microscope and the images were quantified and analyzed using Image J software (NIH, Bethesda, MD) (46).

### Western blotting

Primary mouse podocytes were isolated according to published protocols (18, 47). Briefly, kidneys were collected from 8 to 12 week old mice, mashed with cold PBS and sequentially passed through test sieves (Retsch, Newtown, PA) with pore diameters of 180, 75, and 52 μm, respectively. Glomeruli from the sieve of 52 μm pores were collected in PBS, spun down and resuspended in RPMI 1640 medium containing 10% FBS. Cells were plated on collagen I coated plates for 14 days. Subsequently, the adherent cells were trypsinized and filtered with 40 μm strainer. Filtered cells were spun down and seeded on collagen I coated 6-well plates. Subsequently, cells were washed with ice-cold PBS and lysed using cold lysis buffer (RIPA containing EDTA and EGTA, Boston Bioproducts, Ashland, MA) supplemented with protease inhibitor (Roche Life Science, Indianapolis, IN) and phosphatase inhibitor (Sigma). Cell lysates were incubated on ice for 30 min (with intermittent vortexing) and centrifuged using a tabletop microcentrifuge at 13,000 rpm for 15 min at 4^°^C. Supernatants were carefully transferred to new microcentrifuge tubes and protein concentrations were determined by Bradford assay (Bio-Rad). Equal amounts of protein from each sample were loaded to NuPAGE Novex 4–12% BisTris gels (Life Technologies) and transferred to an Immobilon-P PVDF membrane (EMD Millipore, Billerica, MA). The membranes were blocked with 5% BSA (Thermo Fisher Scientific) in TBS/Tween-20 (0.05%) (Boston Bioproducts, Ashland, MA). Membranes were then incubated with primary antibodies at 4°C overnight. Membranes were washed with TBS containing Tween 20, followed by incubation with a secondary antibody conjugated to horseradish peroxidase (Promega) for 1 h at room temperature. Blots were developed with Super- Signal West Pico chemiluminescent substrate (Thermo Fisher Scientific) and captured using X-ray films (Kodak, Rochester, NY) using an AX-700LE film processor (Alphatek, Houston, TX).

### Quantitative RT-PCR

MicroRNA gene expression analysis was performed using quantitative reverse transcription polymerase chain reaction (qRT-PCR) as previously described (18). Briefly, total RNA or miRNA fractions were isolated from mouse kidney tissue or from mouse podocytes using miRNeasy Mini Kit (Qiagen, Valencia, CA) according to the manufacturer provided protocol and total RNA concentration was measured using NanoDrop (Thermo Fisher Scientific). Isolated RNA (0.5–2.0 μg) was used as template for cDNA synthesis using a TaqMan ™ Advanced miRNA cDNA Synthesis Kit (Thermo Fisher Scientific). qRT-PCR was performed using CFX96 Real-time System (Bio-Rad) and the following TaqMan Gene expression assays (Thermo Fisher Scientific) were used: miR-146a (A25576, ID: 478399_mir), miR361 (A25576, ID: 478056_mir), and Gapdh (4331182, ID: Mm99999915_g1). For analysis, the fold-change in mRNA levels between various groups was determined after normalizing each mRNA expression level with Gapdh [2^(−ΔΔCt)^ method]. The fold-change in miR-146a levels between various groups was determined after normalizing the miR-146a expression level in each group with levels of miR361.

### Lipopolysaccharide-induced proteinuria mouse model

A model of transient proteinuria was used in the wild type C57B/L6 and the Pod-miR-146a^–/–^ mice according to published protocols (48). Briefly, WT and Pod-miR146a^–/–^ male mice (*n* = 5) were divided into two groups and given intraperitoneal (i.p.) injection of phosphate-buffered saline (PBS) or lipopolysaccharide (LPS) at 5 mg/kg (Sigma-Aldrich, #L3024). Spot urine samples were collected at 0, 24, and 72 h post i.p. injection for evaluation of albumin to creatine ratio (ACR). Some animals were sacrificed at 24 h after LPS injection for histologic analysis of kidney tissue. These data are representative of 3 independent experiments.

### Streptozotocin-induced hyperglycemia

A type I diabetes model of inducing hyperglycemia was used in the wild type C57B/L6 and the Pod-miR-146a^–/–^ mice according to published protocols (18, 25). Briefly, 8–10-week-old male mice were administered two doses of streptozotocin (STZ, 125 mg/kg body weight) (Sigma) in 50 mM sodium citrate buffer, pH 4.5, intraperitoneally (i.p.) on days 1 and 4. Glucose levels from tail blood were measured with an AccuCheck glucometer (Roche Life Science). Animals with glucose levels >400 mg/dl on two consecutive measurements were regarded as hyperglycemic. The mice received no insulin during the study period. Urinary albumin and creatinine were analyzed prior to STZ administration and subsequently at intervals shown in the graph. Erlotinib (37 mg/kg) was prepared in saline solution containing 1% Tween-20, 25% Kolliphor, and 2.5% DMSO and was administered intraperitoneally every other day starting at week 4 post-STZ to a group of mice, according to literature protocols (31, 49, 50). Kidneys were harvested and processed for histological and ultrastructural analyses after 16 weeks post-STZ administration.

### Statistical analysis

Data were analyzed using Excel (Microsoft, Redmond, WA) and Prism (GraphPad, San Diego, CA) softwares and data were analyzed using one-way ANOVA, two-way ANOVA, or Student’s *t*-test, where appropriate. *p* < 0.05 was considered statistically significant.

## Data availability statement

The original contributions presented in this study are included in the article/Supplementary material, further inquiries can be directed to the corresponding author.

## Ethics statement

The animal study was reviewed and approved by Institutional Animal Care and Use Committee (IACUC) at Rush University Medical Center.

## Author contributions

VG managed the project and coordinated author activities. XL, IV, HW, and TG designed and performed experiments including *in vivo* assays. AR, VV, and HF helped with tissue acquisition and analyses. XL, RWH, and MA performed western blot analyses. XL, HW, and MA performed statistical analysis. VG, TG, and MA co-wrote the manuscript with input from all authors.

## Supplementary material

Supplementary Figure 1 Representative confocal microscopy images of immunofluorescently labeled glomeruli from WT (top panels) and Pod-miR146a (bottom panels) mice treated with vehicle alone (Control), with STZ and vehicle (STZ) or with STZ and erlotinib (STZ + Erl). Tissue sections were imaged after staining with DAPI (nuclear marker, blue) and antibodies against ErbB4 (red) and Synaptopodin (green). Colocalization between ErbB4 and synaptopodin is apparent as yellow. Scale bar, 50 μm.

Supplementary Figure 2 Representative confocal microscopy images of immunofluorescently labeled glomeruli from WT and Pod-miR146a mice treated with vehicle alone (Control), with STZ and vehicle (STZ) or with STZ and erlotinib (STZ + Erl). Tissue sections were imaged after staining with DAPI nuclear marker) and anti-Synaptopodin antibody (Synpo, podocyte marker) (as indicated) to identify and manually count podocytes. Scale bar, 50 μm. **(B)** Bar graphs showing quantification of podocyte numbers per glomerular section from at least five glomeruli from three independent samples per group. Data shown are mean ± SEM. Statistics were performed using two-way ANOVA. **p* < 0.05; ^****^*p* < 0.001; ns, no significant difference.

Supplementary Figure 3 Original immunoblot images for the data presented in Figure 7.
