# Supplementary figures and images for: Podocyte-specific deletion of miR-146a increases podocyte injury and diabetic kidney disease
Source: Front Med (Lausanne). 2022 Aug 18;9:897188. doi: 10.3389/fmed.2022.897188 (PMC9433550; doi:10.3389/fmed.2022.897188)

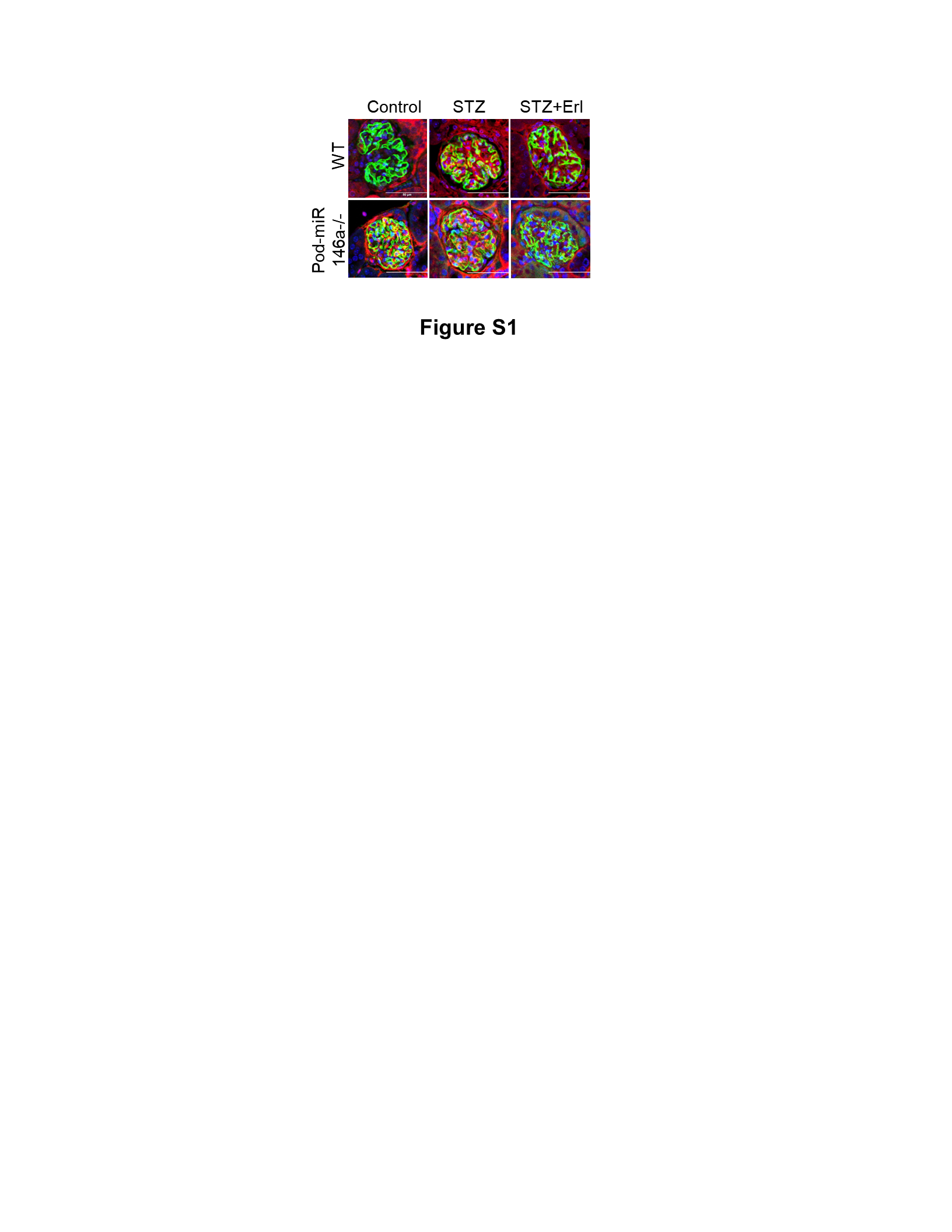

Supplement: Supplementary Figure 1 — Representative confocal microscopy images of immunofluorescently labeled glomeruli from WT (top panels) and Pod-miR146a (bottom panels) mice treated with vehicle alone (Control), with STZ and vehicle (STZ) or with STZ and erlotinib (STZ + Erl). Tissue sections were imaged after staining with DAPI (nuclear marker, blue) and antibodies against ErbB4 (red) and Synaptopodin (green). Colocalization between ErbB4 and synaptopodin is apparent as yellow. Scale bar, 50 μm. [file Image_1.tif]

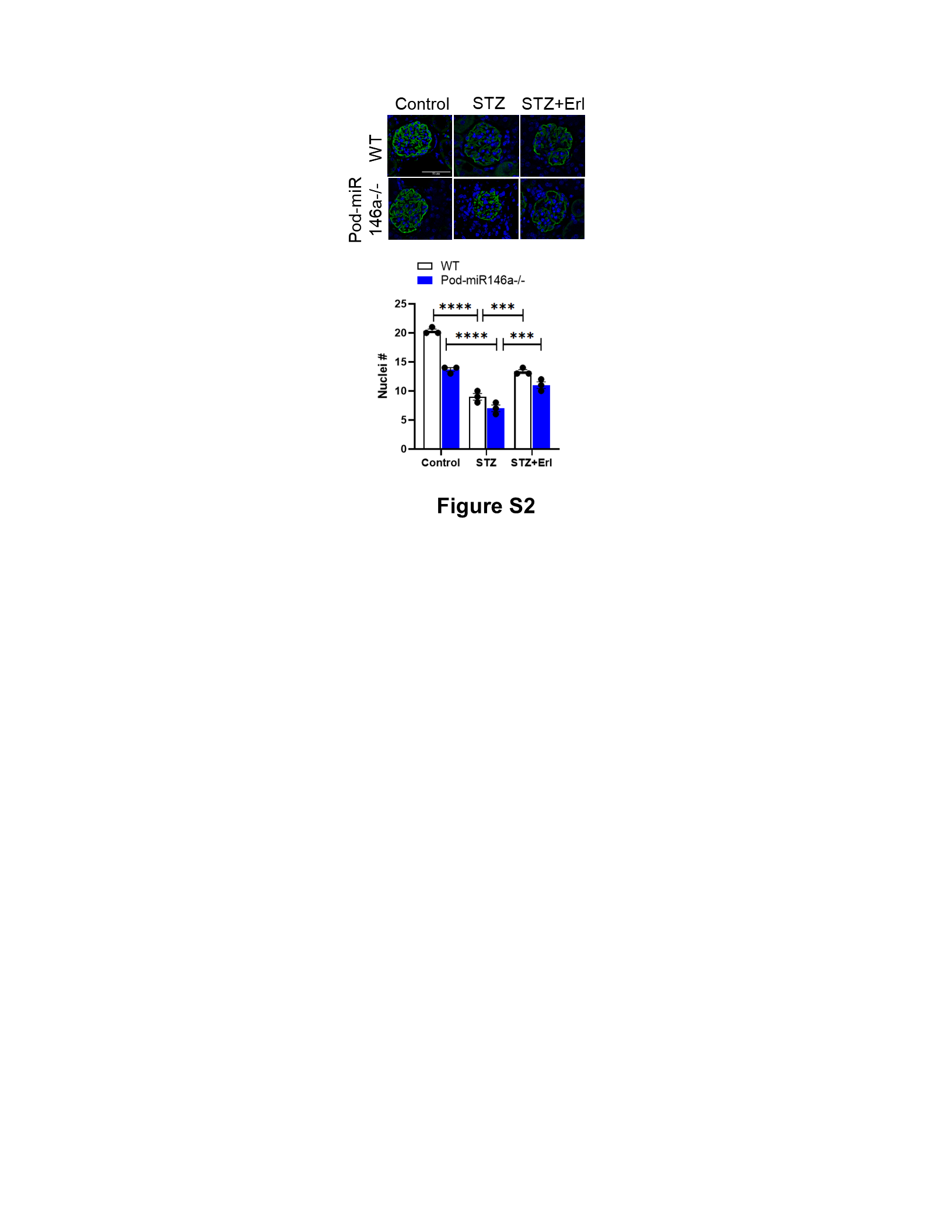

Supplement: Supplementary Figure 2 — Representative confocal microscopy images of immunofluorescently labeled glomeruli from WT and Pod-miR146a mice treated with vehicle alone (Control), with STZ and vehicle (STZ) or with STZ and erlotinib (STZ + Erl). Tissue sections were imaged after staining with DAPI nuclear marker) and anti-Synaptopodin antibody (Synpo, podocyte marker) (as indicated) to identify and manually count podocytes. Scale bar, 50 μm. (B) Bar graphs showing quantification of podocyte numbers per glomerular section from at least five glomeruli from three independent samples per group. Data shown are mean ± SEM. Statistics were performed using two-way ANOVA. *p < 0.05; ****p < 0.001; ns, no significant difference. [file Image_2.tif]

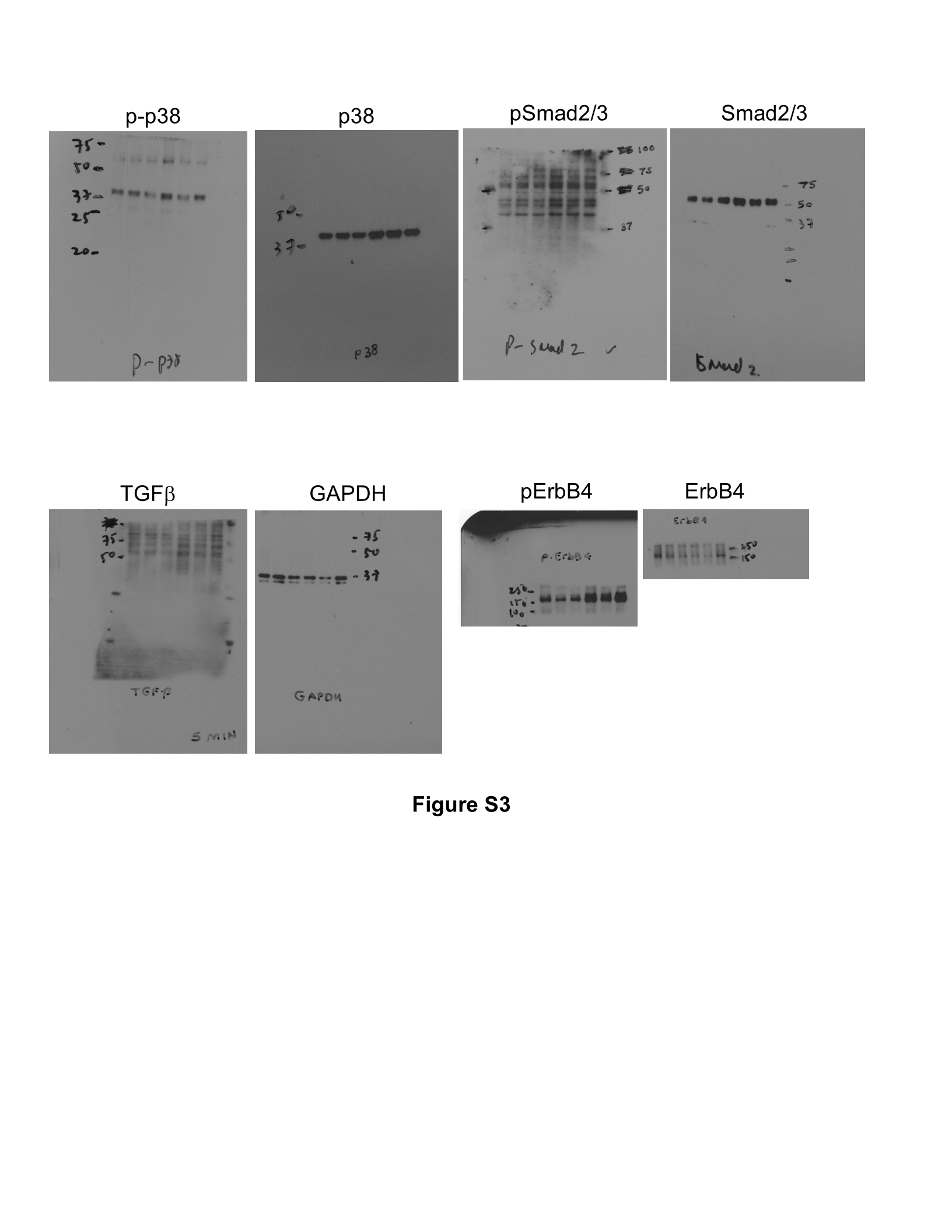

Supplement: Supplementary Figure 3 — Original immunoblot images for the data presented in Figure 7. [file Image_3.tif]
